# PEBP4 deficiency aggravates LPS-induced acute lung injury and alveolar fluid clearance impairment via modulating PI3K/AKT signaling pathway

**DOI:** 10.1007/s00018-024-05168-5

**Published:** 2024-03-13

**Authors:** Qiao-qing Shi, Yong-hong Huang, Yu-fei Li, Shuang-yan Zhen, Yan-hong Li, Jia-yi Huang, Jia-yang Wang, Xiao-yan Zhou

**Affiliations:** 1 Department of Pathophysiology, School of Basic Medical Sciences, Jiangxi Medical College, Nanchang University, 461 BaYi Road, Nanchang, 330006 Jiangxi P.R. China; 2 Department of Science and Education, Jiangxi Chest Hospital, Nanchang, 330006 China; 3 Sino-German Joint Research Institute, Nanchang University, Nanchang, 330047 China; 4 Department of Forensic Medicine, School of Basic Medical Sciences, Jiangxi Medical College, Nanchang University, Nanchang, 330006 China; 5 School of Basic Medical Sciences, Nanchang University, Nanchang, 330103 China; 6 School of Stomatology, Nanchang University, Nanchang, 330103 China; 7 Jiangxi Province Key Laboratory of Tumor Etiology and Molecular Pathology, Nanchang, 330006 China

**Keywords:** Acute lung injury, Phosphatidylethanolamine binding protein 4 (PEBP4), Inflammatory response, Alveolar fluid clearance, PI3K/AKT signaling pathway

## Abstract

Acute lung injury (ALI) is a common clinical syndrome, which often results in pulmonary edema and respiratory distress. It has been recently reported that phosphatidylethanolamine binding protein 4 (PEBP4), a basic cytoplasmic protein, has anti-inflammatory and hepatoprotective effects, but its relationship with ALI remains undefined so far. In this study, we generated PEBP4 knockout (KO) mice to investigate the potential function of PEBP4, as well as to evaluate the capacity of alveolar fluid clearance (AFC) and the activity of phosphatidylinositide 3-kinases (PI3K)/serine-theronine protein kinase B (PKB, also known as AKT) signaling pathway in lipopolysaccharide (LPS)-induced ALI mice models. We found that PEBP4 deficiency exacerbated lung pathological damage and edema, and increased the wet/dry weight ratio and total protein concentration of bronchoalveolar lavage fluid (BALF) in LPS-treated mice. Meanwhile, PEBP4 KO promoted an LPS-induced rise in the pulmonary myeloperoxidase (MPO) activity, serum interleuin (IL)-1β, IL-6, and tumor necrosis factor (TNF)-α levels, and pulmonary cyclooxygenase-2 (COX-2) expression. Mechanically, PEBP4 deletion further reduced the protein expression of Na^+^ transport markers, including epithelial sodium channel (ENaC)-α, ENaC-γ, Na,K-ATPase α1, and Na,K-ATPase β1, and strengthened the inhibition of PI3K/AKT signaling in LPS-challenged mice. Furthermore, we demonstrated that selective activation of PI3K/AKT with 740YP or SC79 partially reversed all of the above effects caused by PEBP4 KO in LPS-treated mice. Altogether, our results indicated the PEBP4 deletion has a deterioration effect on LPS-induced ALI by impairing the capacity of AFC, which may be achieved through modulating the PI3K/AKT pathway.

## Introduction

Acute lung injury (ALI) is a common critical pathological process with an extremely high mortality rate [1], which is caused by various pulmonary and extra-pulmonary factors other than cardiogenic ones. Its main feature is the damage to the barrier composed of alveolar epithelial cells (AECs) and capillary endothelial cells, thereby leading to the accumulation of protein-rich fluid in the alveolar space, which can result in severe hypoxemia and acute respiratory failure [2]. Currently, the clinical treatments for ALI mainly include lung-protective mechanical ventilation [3], drug therapy [4], mesenchymal stem cell transplantation [5], etc. Howbeit, there are problems such as poor efficacy and expensive treatment costs, especially lacking in specific treatments by far. Therefore, it is urgent to elucidate the pathogenesis of ALI and develop its novel intervention targets and drugs.

It is crucial for the prognosis of ALI to remove pulmonary edema fluid in a timely and effective manner and to improve the lung’s oxygenation ability [6]. Epithelial sodium channel (ENaC), a heterotetramer protein composed of two α subunits, one β subunit, and one γ subunit, plays a critical role in the process of alveolar fluid clearance (AFC) [7]. Generally, sodium ion in alveolar space is transported into AECs via ENaC located on the apical membrane of AECs, and then into the pulmonary interstitium with the help of Na,K-ATPase located on the basement membrane of AECs. During this process, sodium ion transportation across membrane creates an osmotic gradient that drives the reabsorption of edematous fluid from alveolar space into pulmonary interstitium, and then alleviates pulmonary edema through venous and lymphatic back-flow [6].

Phosphatidylethanolamine binding protein 4 (PEBP4) is the most important member of the PEBPs family, and is a cytoplasmic alkaline protein with multiple biological functions and high expression in mammals [8]. Interestingly, several reports reveal that PEBP4 is also a secreted protein [8, 9]. PEBP4 has been reported to have anti-apoptotic function and is associated with the occurrence and development of various tumors [10]. In addition, PEBP4 is closely related to acute liver injury and liver fibrosis on account of its anti-inflammatory and liver-protective effects [11, 12]. PEBP4 has been also identified as a new-found marker of type II AECs [13], whose dysfunction is involved in the occurrence and progression of ALI. Moreover, the serum PEBP4 level is negatively correlated with the concentration of sodium ion [14], and AFC relies mainly on the ENaC-mediated Na^+^ transport of type II AECs. Accordingly, it is speculated that PEBP4 may regulate the expression of Na^+^ transporter and involve in AFC in ALI .

phosphatidylinositide 3-kinases (PI3K)/serine-theronine protein kinase B (PKB, also known as AKT) signaling pathway can effectively regulate inflammatory responses and plays an important role in ALI. In some cases, the phosphorylation level of this pathway is significantly inhibited along with a reduced ENaC expression and serious pulmonary edema when ALI [15]. Intriguingly, several recent research have revealed the relationship between PEBP4 and the PI3K/AKT pathway. PEBP4 is found to act as a scaffold protein for AKT/mammalian target of rapamycin (mTOR) signal transduction, and PEBP4 knock-down disturbs the interaction between AKT and mTOR [16]. In lung cancer cells, overexpression of PEBP4 promotes the AKT and mTOR phosphorylation, while PEBP4 knock-down shows the opposite result [17]. These findings may pose an interesting question whether PEBP4 regulates AFC to affect ALI via the PI3K/AKT signaling pathway.

In the present study, we established a PEBP4 knockout (KO) mouse model to investigate the influence of PEBP4 on lipopolysaccharide (LPS) -induced ALI and its potential mechanism involved. Our results showed PEBP4 deficiency exacerbated lung damage and edema, and promoted a rise in the pulmonary myeloperoxidase (MPO) activity, serum interleukin(IL)-1β, IL-6, and tumor necrosis factor (TNF)-α levels, and pulmonary cyclooxygenase-2 (COX-2) expression in the LPS-challenged mice. In terms of its mechanism, PEBP4 deletion further reduced the protein expression of AFC-related markers including ENaC-α, ENaC-γ, Na,K-ATPase α1, and Na,K-ATPase β1, and enhanced the suppression of PI3K/AKT signaling pathway in ALI models, which were partially rectified by selective activation of PI3K/AKT pathways with 740YP or SC79.

## Materials and methods

### Reagents

LPS (*Escherichia coli*, O55:B5) was obtained from Sigma-Aldrich (St. Louis, MO, USA). 740YP and SC79 were acquired from MedChem Express (Shanghai, China). The MPO detection kit was purchased from Nanjing Jiancheng Institute of Biological Engineering (Nanjing, China). The enzyme-linked immunosorbent assay (ELISA) kits for TNF-α, IL-1β and IL-6 were provided by Abclone (Wuhan, China), Yunclone Technology (Wuhan, China) and Invitrogen (Carlsbad, CA, USA), respectively. The PEBP4 antibody was from RayBiotech (Atlanta, USA). Antibodies for GAPDH, COX-2, ENaC-β, ENaC-γ, Na, K-ATPase α1, Na, K-ATPase β1 and AKT were purchased from Proteintech (Chicago, IL, USA). Antibodies for ENaC-α, PI3K were produced by Affinity (Guangzhou, China). The p-PI3K antibody was generated from Abmart (Shanghai, China). The antibody for p-AKT was generated by Cell Signaling Technology (Boston, Massachusetts, USA).

### Animals

The *PEBP4*^flox/+^ mice and the *CAG-Cre*^+^ mice with C57BL/6 N background were provided by Cyagen Biosciences (Guangzhou, China). C57BL/6 N wild type (WT) mice were purchased from GemPharmatech (Nanjing, China). All mice were housed in a specific pathogen-free animal facility with free access to food and water. All animal care and experimental methods were conducted in accordance with the National Institutes of Health Guidelines for the Care and Use of Laboratory Animals, and the protocols were approved by the Animal Care and Use Committee of Nanchang University [SYXK(Gan)2021-0004]. All mice used in this study were at the age of 6–8 weeks and the weight of 18–22 g, and randomly chosen for the experiments.

### Generation and identification of PEBP4 KO mice

To generate PEBP4 KO mice, we firstly crossed the *PEBP4*^flox/+^ mice with each other to obtain *PEBP4*^flox/flox^ mice. Subsequently, the *PEBP4*^flox/flox^ mice were crossed with *CAG-Cre*^+^ mice to generate *PEBP4*^flox/+^;*CAG-Cre*^+^ mice. Lastly, the *PEBP4*^*−*/−^ mice, i.e. PEBP4 KO mice, were produced by mating the *PEBP4*^flox/+^;*CAG-Cre*^+^ mice each other. Age-, gender- and weight-matched mice were used in the subsequent experiment.

The mouse PEBP4 KO was identified by polymerase chain reaction (PCR) analysis and western blotting analysis, respectively. DNA from the mouse tail was extracted by a base cleavage method, then the DNA was used as a template for PCR assay. A 38-cycle (94℃ 30 s, 60℃ 35 s, 72℃ 35 s) was applied in the PCR assay. Agarose gel electrophoresis was used to separate the PCR products after PCR assay. The details about primers and their corresponding PCR products were attached in Table 1. The target band for WT mice was located at 188 bp, while the target band for PEBP4 KO mice was present at 277 bp. Genotyping of *CAG-Cre*^+^ mice was performed in accordance with the standard protocol of the Jackson Laboratory.

**Table 1:** Sequences used for PCR

No	Primer name	Primer sequence (5’-3’)	Band size
1	loxp-F	GATCCTGGAGCTACTGAAAGCACTGAG	Flox = 251 bp WT = 188 bp
	loxp-R	GCTATTTACACCACCATGCCCTGC	
2	PEBP4 del-F	GATCCTGGAGCTACTGAAAGCACTGAG	PEBP4 KO = 277 bp
	PEBP4 del-R	ACAACCAGAAGGATGAAATCGGAAAC	
3	GAPDH-F	AGGTCGGTGTGAACGGATTTG	GAPDH = 123 bp
	GAPDH-R	TGTAGACCATGTAGTTGAGGTCA	

### Experimental grouping

For the experiment of LPS-induced ALI alone, WT mice and PEBP4 KO mice were randomly divided into two groups (*n* = 12 per group) as follows: (1) control group, in which mice received equal volume of phosphate buffer solution (PBS) by intratracheal instillation; (2) LPS group, in which mice received 5 mg/kg LPS by intratracheal instillation. The route of LPS or PBS administration was modified according to the reference [18]. Briefly, after sufficient anesthesia with 10% chloral hydrate, place the mouse in prone position on a table, and then super-extend the neck in an about 90°angle relative to the table. Hold the tongue with forceps to straighten the throat for easier intubation conditions. Gently insert the catheter in the vertical direction along the tongue’s root. Place a cold-light source to help visualize the vocal chords and aim for the trachea. Then, insert the catheter approximately 10 mm into the trachea. Ensure that the insertion is not too deep as this will result in unilateral instillation of fluid into the right or left main bronchus. Finally, inject LPS or PBS using a pipette. After injection, keep the mouse’s upper body in an upright position for 30 s to avoid leakage of the fluid from the trachea. For the experiment of PI3K or AKT activator combined with LPS treatment, WT mice and PEBP4 KO mice were randomly divided into three groups (*n* = 12 per group) as follows: (1) LPS group, in which mice received 5 mg/kg LPS by intratracheal instillation; (2) LPS + 740YP (PI3K activator) group, in which mice received 300 mg/kg of 740YP by intraperitoneal (i.p.) injection 30 min before 5 mg/kg LPS administration; (3) LPS + SC79 (AKT activator) group, in which mice received 5 mg/kg of SC79 by i.p. injection 30 min before 5 mg/kg LPS administration. Age- and weight-matched male mice were used in the ALI model.

### Histological examination

After 8 h of LPS stimulation, the right upper lobe of lung was collected, and then fixed with 4% paraformaldehyde solution, embedded in paraffin, and sectioned 4–6 μm for histological analysis. Samples were deparaffinized and rehydrated prior to staining. Hematoxylin and eosin (H&E) was performed according to standard procedures using an H&E staining kit (Servicebio, China). Morphological changes were assessed under light microscopy (Olympus, Japan), and the grade of lung injury was evaluated using a semi-quantitative scoring system as previously described [15]. The items mainly included alveolar exudate, interstitial edema, alveolar hemorrhage, and inflammatory cell infiltration. The corresponding score per item extent was as follows: 0 = no injury; 1 = minor injury (25%); 2 = moderate injury (50%); 3 = severe injury (75%); 4 = very severe injury (almost 100%). The result for each item was graded from 0 to 4, and the four items were summed to represent the lung injury total score (0–16). At least three high power and non-overlapping fields per mouse were viewed and evaluated based on the above scoring system, and the final scores were averaged.

### Small animal micro-CT imaging

After 8 h of modeling, mice were anesthetized and fixed. Put the mice into the table of a small animal micro computed tomography (micro-CT) machine (Karlsruhe, Germany) to scan their chest, and then acquire the CT images by a Bruker Skyscan 1278 system. Mice in each group were scored for lung CT severity [19]. The extent of involvement of each lobe was scored separately based on lung CT pictures. No = 0 points, 1–5% =1 point, 5–25% =2 points, 25–50%=3 points, 50–75%= 4 points, > 75%=5 points. All scores were summed to represent the total score of lung CT severity.

### Measurement of lung wet/dry weight ratio

After the mice were sacrificed, the right middle lobes of lungs were first rinsed in precooling PBS to wash off the surface blood, and then the water on the surface was wiped off with a filter paper towel. Then, its weight was seen as the wet weight. After that, baked it at 55℃ for 72 h in a thermostatic oven. When the weight kept stable, this weight was considered as the dry weight. The wet/dry weight ratio (W/D) = the value of wet weight/dry weight.

### Detection of protein concentration in bronchoalveolar lavage fluid

The broncholaveolar lavage fluid (BALF) from mice was collected according to the previous study [20]. Subsequently, the BALF was determined using a bicinchoninic acid (BCA) protein assay kit according to the manufacturer’s instructions.

### Pulmonary MPO activity measurement

Lung tissues were accurately weighed and homogenized. After that, the homogenized tissue fluid was centrifuged at 30,000 g for 30 min. The pellet was resuspended in potassium phosphate buffer with 0.5% hexadecyltrimethyl ammonium bromide. The Samples were centrifuged at 20,000 g for 15 min at 4℃ and the supernatants were collected. The myeloperoxidase (MPO) activity in lung was evaluated by measuring absorbance at 460 nm. Results were presented as units of MPO per gram of wet lung tissue.

### ELISA assay

Serum samples were obtained by centrifugation of blood samples. The levels of of TNF-α, IL-1β, and IL-6 in serum was detected using ELISA kits. The details about the detection was in accordance with the manufacturer’s instructions. Thereafter, the concentrations of these cytokines were evaluated according to the absorbance at 450 nm.

### Western blotting analysis

Western blotting was performed according to standard protocols. Briefly, total protein was extracted from lung tissues according to the instructions of the protein extraction kit. The protein concentration was then detected with a BCA protein assay kit. Proteins were separated using 8% or 12% polyacrylamide gels and transferred onto polyvinylidene fluoride (PVDF) membranes. The membranes were blocked with 5% bovine serum albumin (BSA) or 5% fat-free milk dissolved in Tris-buffered saline plus 0.1% Tween 20 (TBST) for 3 h at room temperature followed by incubation with the corresponding specific primary antibodies (GAPDH, PEBP4, COX-2, ENaC-α, ENaC-β, ENaC-γ, Na, K-ATPase α1, Na, K-ATPase β1, AKT, PI3K, p-AKT, and p-PI3K) overnight at 4℃. The following day, membranes were washed with TBST for three times and then incubated with horseradish peroxidase- conjugated suitable secondary antibodies (1:10,000 dilution). Finally, bands were visualized with enhanced chemiluminescence and a gel imaging system (Bio-Rad, Heracles, CA, USA) and quantitative analysis was performed using ImageJ software (NIH, Bethesda, MD, USA).

### Statistical analysis

All data in this study were expressed as means ± SD. The differences between groups were performed using one-way analysis of variance (ANOVA) or an unpaired *t*-test. A *p* < 0.05 was considered to be statistically significant. All statistical analyses and plots were performed using GraphPad Prism 9.3.1 software.

## Results

### PEBP4 KO establishment and its phenotype analysis

**Fig. 1:**
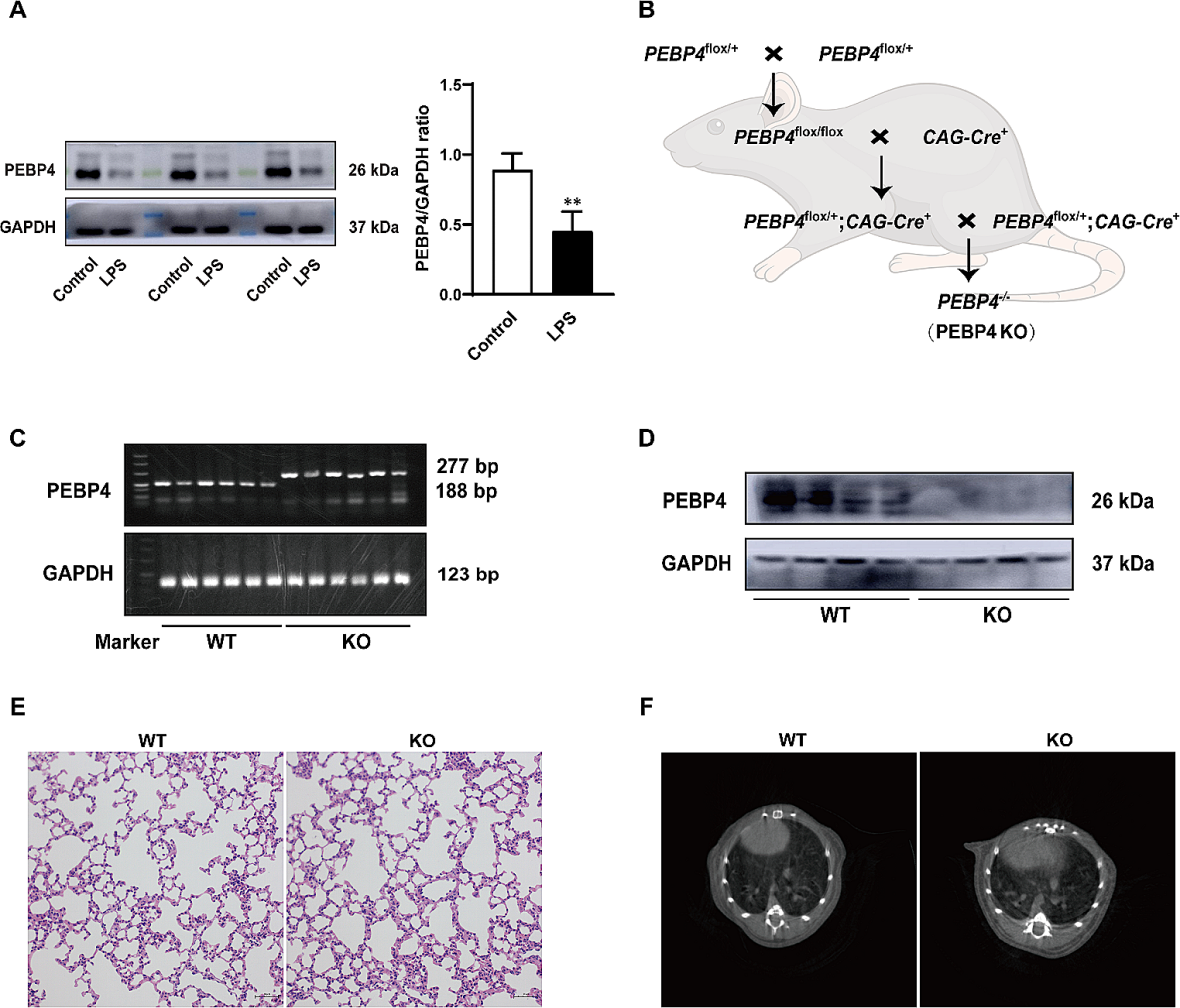
Establishment of PEBP4 knockout (KO) mice and their phenotype analysis. (**A**) PEBP4 protein expression was detected by western blotting in the lung tissues of the WT control and LPS mice. (**B**) Schematic diagram for PEBP4 KO mice (*PEBP4*^−/−^) production using the breeding technique. (**C**) Identification of PEBP4 KO at the DNA level was detected by PCR and agarose gel electrophoresis. (**D**) Identification of PEBP4 KO at the protein level was tested by western blotting. (**E**) Histological changes of lung tissues by H&E staining (×200 magnification). (**F**) Morphological changes of lung tissues based on small animal micro-CT imaging. Data are presented as mean ± SD values (*n* = 3). **p < 0.01 compared to WT Control group

To elucidate the relationship between PEBP4 expression and ALI, an ALI model induced by LPS was established using WT mice. Then the protein expression levels of PEBP4 in the lung tissues of the control group and the LPS group were detected by western blotting. The results showed that PEBP4 expression was significantly down-regulated in the LPS group (Fig. 1A). To further explore the effect and mechanism of PEBP4 in ALI, we used CAG-Cre^+^ mice and PEBP4^flox/+^ mice to construct PEBP4 KO mice by breeding technique (Fig. 1B). The knockout of PEBP4 in lung tissue was confirmed by PCR (Fig. 1C) and western blotting (Fig. 1D). These results showed PEBP4 KO mice were successfully generated. Regrettably, the data from both H&E staining (Fig. 1E) and small animal micro-CT imaging (Fig. 1F) showed that there was no significant difference in lung morphology after PEBP4 deletion.

### PEBP4 deficiency aggravates LPS-induced acute lung injury

**Fig. 2:**
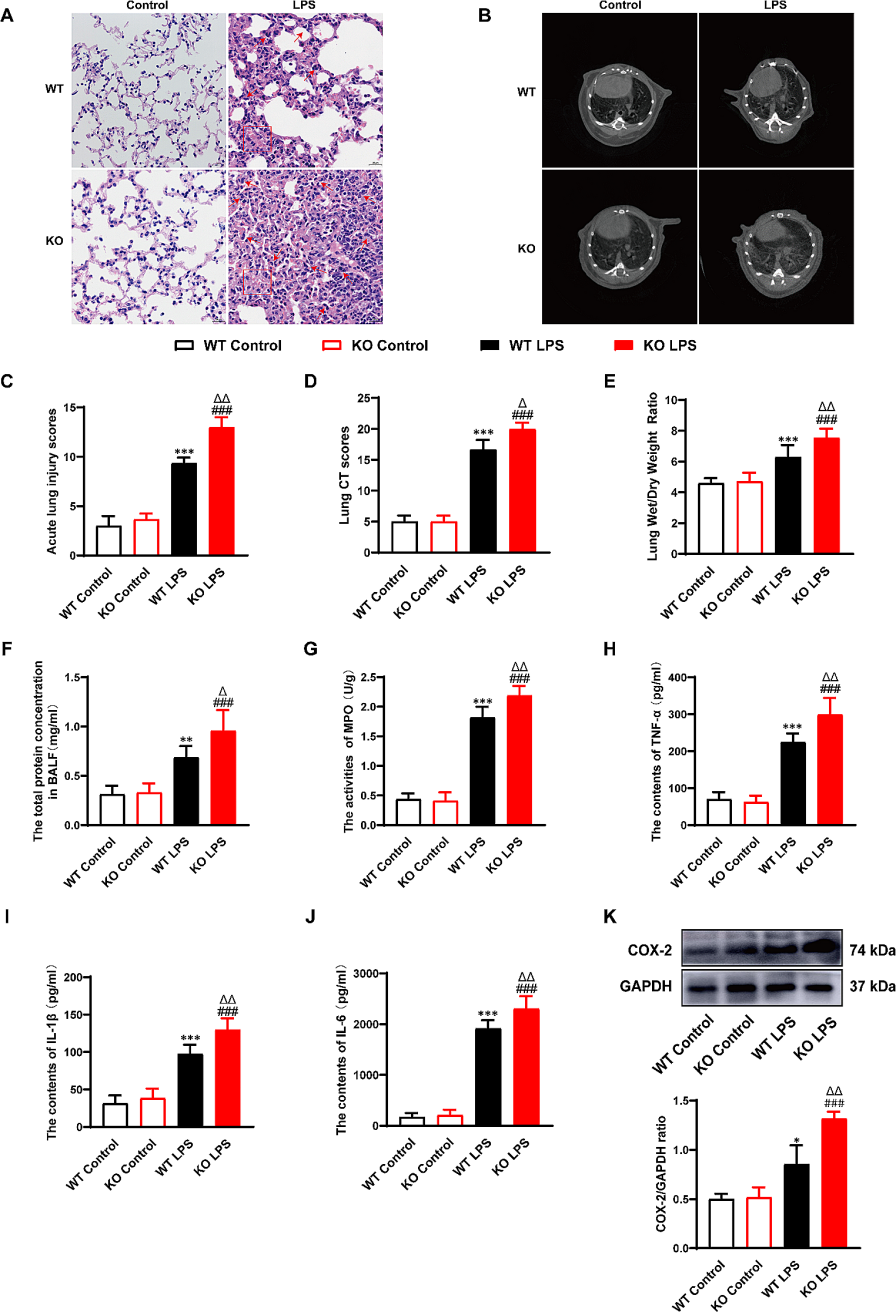
PEBP4 deficiency aggravated LPS-induced ALI. (**A**) Histological changes of lung tissue (H&E staining, ×400), ↑ for inflammatory cell infiltration, □ for Hemorrhage, congestion. (**B**) Structural changes of lung tissue based on small animals micro-CT imaging. (**C**) The injury scores of H&E were quantified. (**D**) The injury scores of CT were quantified. (E) Lung wet/dry weight ratio (*n* = 6). (**F**) Detection of total protein concentration in BALF (*n* = 6). (**G**) MPO activities were tested in lung tissue (*n* = 6). (**H**-**J**) The levels of TNF-α, IL-1β and IL-6 in serum were detected by ELISA (*n* = 6). (**K**) Western blotting was used to examine the expression of COX-2 (*n* = 3). Data are presented as mean ± SD values. *p < 0.05, **p < 0.01 and ***p < 0.001 vs. the WT control group; ^###^*p* < 0.001 compared to the KO control group; ^Δ^*p* < 0.05 and ^ΔΔ^*p* < 0.01 compared to the WT LPS group

To further observe whether PEBP4 deletion might affect the progression of ALI, we applied LPS-induced ALI models in both WT mice and PEBP4 KO mice. As shown in Fig. 2A, compared with the control groups, the LPS groups in both WT mice and PEBP4 KO mice presented obvious structural alternations, manifested by damaged alveolar walls, collapsed alveoli merged into lung bullae, bleeding, edema, and lung consolidation. More importantly, in the LPS groups, these pathological changes in the PEBP4 KO mice were more significant than those in the WT mice. And a similar phenomenon was observed in the result of lung micro-CT imaging (Fig. 2B). Furthermore, the scores related to both H&E staining (Fig. 2C) and micro-CT imaging (Fig. 2D) quantitatively confirmed the accelerated role of PEBP4 deficiency in the ALI model. Pulmonary edema is an important characteristic of ALI. Thus we examined W/D ratio of lung tissue and total protein concentration of BALF. Compared to the control groups, both lung tissue W/D ratio (Fig. 2E) and total protein concentration of BALF (Fig. 2F) were significantly increased in the LPS groups. Consistently, in the LPS groups, both W/D ratio and total protein concentration of BALF became higher after PEBP4 KO, indicating pulmonary edema was more prone to form in PEBP4 KO mice. Inflammation is another vital feature of ALI. To evaluate the degree of inflammatory response, the levels of MPO, TNF-α, IL-1β, IL-6 and COX-2 were detected. As expected, in LPS-treated mice, the activity of lung MPO (Fig. 2G), the serum levels of TNF-α (Fig. 2H), IL-1β (Fig. 2I), and IL-6 (Fig. 2J), and the expression of lung COX-2 (Fig. 2K) were noticeably increased, and LPS-treated KO mice had a more obvious rise compared with LPS-treated WT mice (Fig. 2G-K). Altogether, the results suggest that PEBP4 plays a critical role in the progression of ALI.

### PEBP4 deficiency exacerbates the disturbance of AFC and the inhibition of PI3K/AKT signaling pathway

**Fig. 3:**
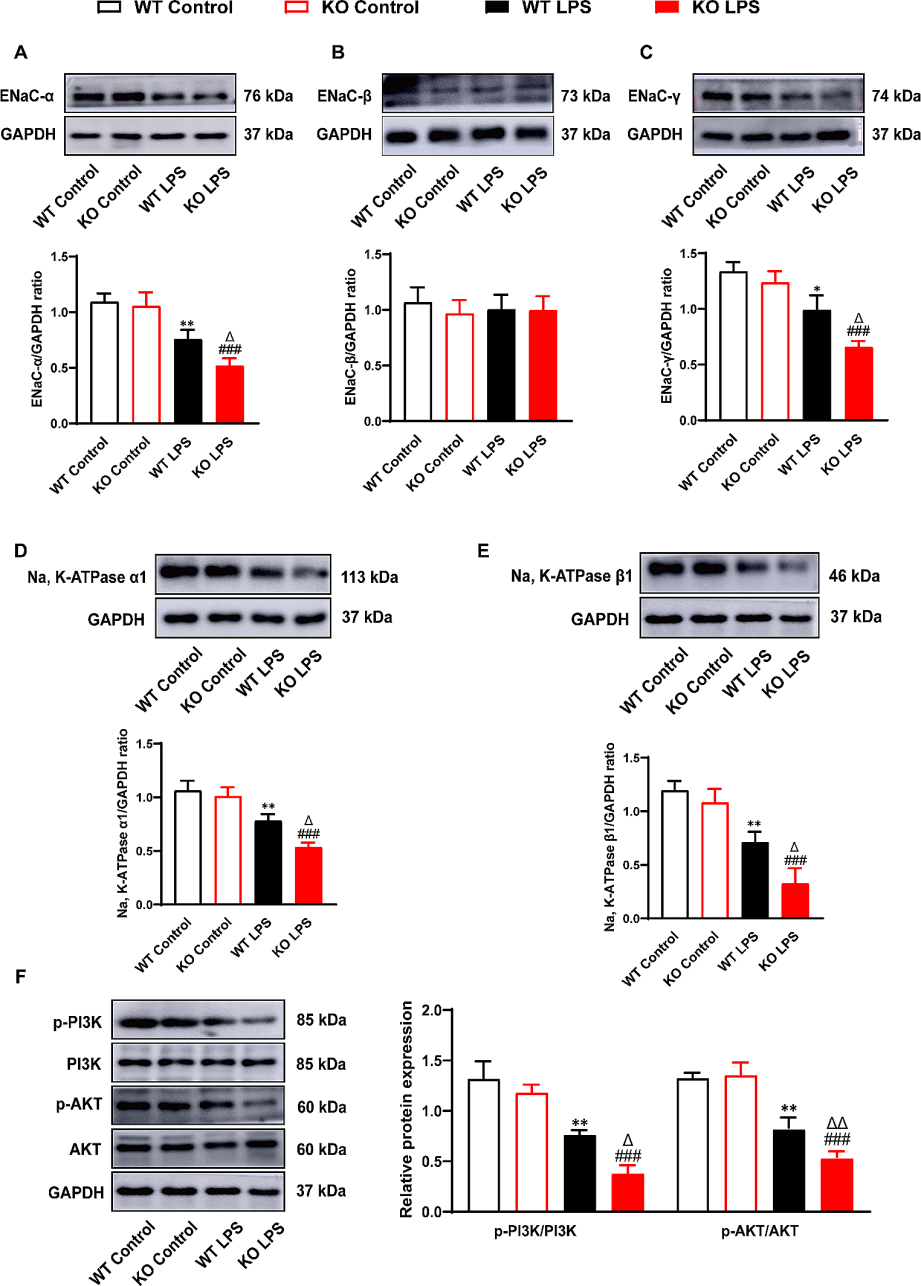
PEBP4 KO exacerbated the disturbance of AFC and suppressed the activation of the PI3K/AKT pathway. Western blotting was employed to examine the expressions of ENaC-α, β, γ, Na, K-ATPase α1, β1, p-PI3K and p-AKT(*n* = 3). Data are presented as mean ± SD values. *p < 0.05 and **p < 0.01 vs. the WT control group; ^###^*p* < 0.001 compared to the KO control group; ^Δ^*p* < 0.05 and ^ΔΔ^*p* < 0.01 compared to the WT LPS group

ENaC and Na, K-ATPase play major roles in AFC. To further elucidate whether PEBP4 deficiency aggravates the disturbance of AFC, these AFC-related proteins were extracted from mice lungs, and then their expressions were measured by western blotting. As presented in Fig. 3, the expression levels of ENaC-α, ENaC-γ, Na, K-ATPase α1, and Na, K-ATPase β1 in the LPS groups were lower than those in the control groups. Likewise, PEBP4 KO further attenuated the expression levels of these proteins in ALI mice. Confusedly, ENaC-β expression showed no significant discrepancy in all of the groups (Fig. 3B). Taken together, these findings indicate that the disturbance of AFC occurred in LPS-induced ALI, and PEBP4 loss could make the disturbance more serious. In addition, we analyzed the activated status of key molecules based on PI3K/AKT pathway. In consistent with previous studies [21], LPS treatment down-regulated the expressions of both p-PI3K and p-AKT (Fig. 3F). Furthermore, the PEBP4 KO mice displayed much lower expression of both p-PI3K and p-AKT compared with the WT mice in ALI (Fig. 3F). The data suggests that the PI3K/AKT signaling pathway could mediate the function of PEBP4 in ALI.

### PI3K/AKT signaling activators specifically reverse the impact of PEBP4 deletion on LPS-induced ALI

**Fig. 4:**
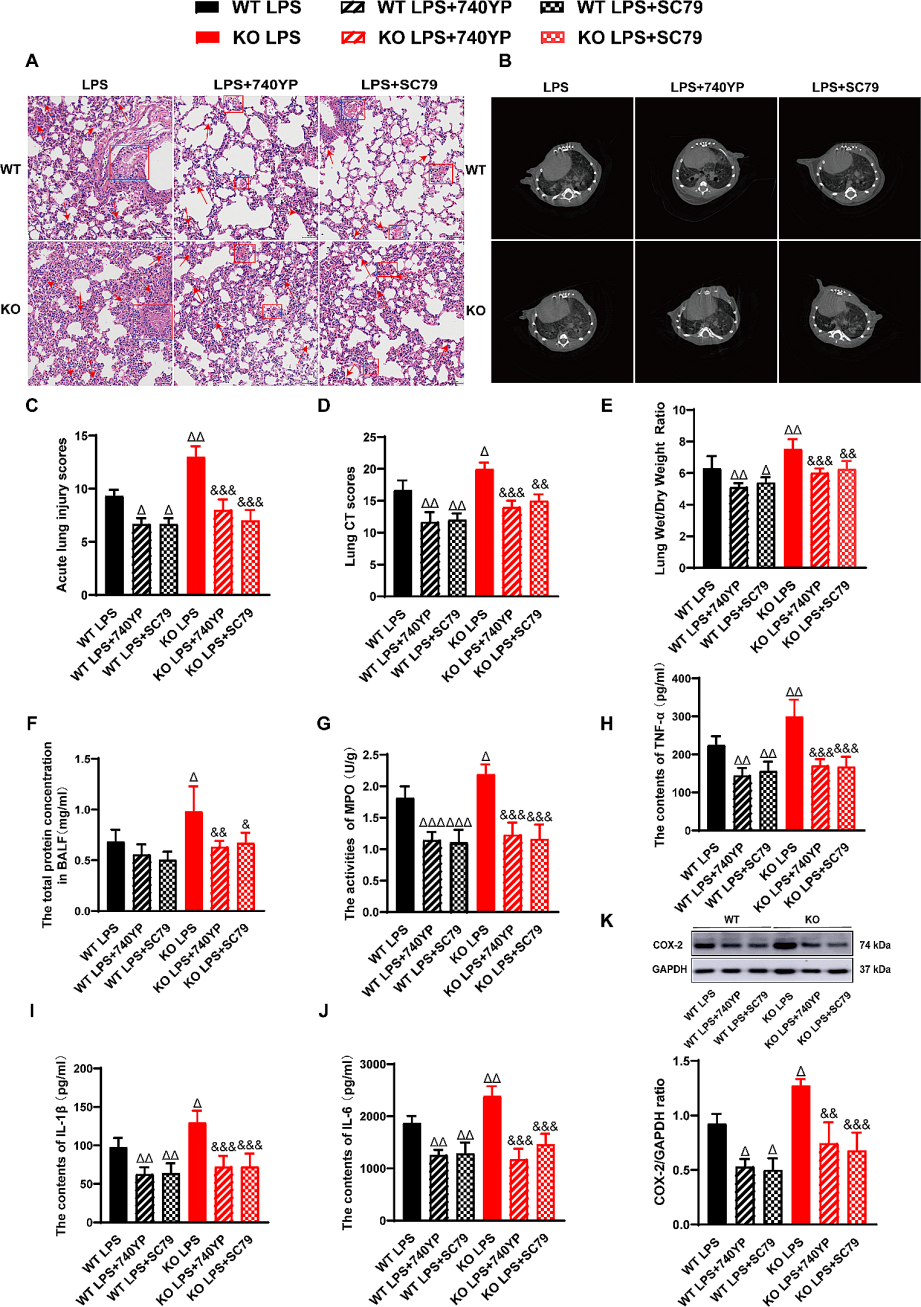
Activated PI3K/AKT signaling partially reversed the impact of PEBP4 deficiency on LPS-induced ALI. (**A**) Histological changes of lung tissue (H&E staining, ×200), ↑ for inflammatory cell infiltration, □ for Hemorrhage, congestion. (**B**) Small animals micro-CT imaging changes of lung tissue. (**C**) The injury scores of H&E staining were quantified (*n* = 3). (**D**) The injury scores of CT were quantified (*n* = 3). (**E**) Lung wet/dry weight ratio (*n* = 6). (**F**) Detection of total protein concentration in BALF (*n* = 6). (**G**) MPO activities were tested in lung tissue (*n* = 6). (**H**, **I**, **J**) The levels of TNF-α, IL-1β and IL-6 in serum were detected by ELISA (*n* = 6). (**K**) Western blotting was used to examine the expression of COX-2 (*n* = 3). Data are presented as mean ± SD values. ^Δ^*p* < 0.05, ^ΔΔ^*p* < 0.01 and ^ΔΔΔ^*p* < 0.001 vs. the WT LPS group; ^&^*p* < 0.05, ^&&^*p* < 0.01 and ^&&&^*p* < 0.001 compared to the KO LPS group

To further clarify the specific mediation of PI3K/AKT signaling in the aggravation of PEBP4 loss to ALI, we examined the effect of PI3K activator 740YP and AKT activator SC79 in LPS-treated PEBP4 KO models. Both H&E staining and CT imaging analysis showed that the two activators could partially reverse the impact of PEBP4 deficiency on the lung structure in ALI (Fig. 4A-B), and their related scores further supported these effects (Fig. 4C-D). In addition, 740YP and SC79 partly rectified the degree of pulmonary edema, characterized by decreased W/D ratio (Fig. 4E) and total protein concentration in BALF (Fig. 4F). 740YP and SC79 also partly reversed the grade of inflammatory response, including reduced lung MPO activity (Fig. 4G), serum levels of TNF-α (Fig. 4H), IL-1β (Fig. 4I), and IL-6 (Fig. 4J), and lung COX-2 expression (Fig. 4K). Collectively, these results further support that PI3K/AKT signaling pathway might mediate the PEPBP’s roles in LPS-induced ALI.

**Fig. 5:**
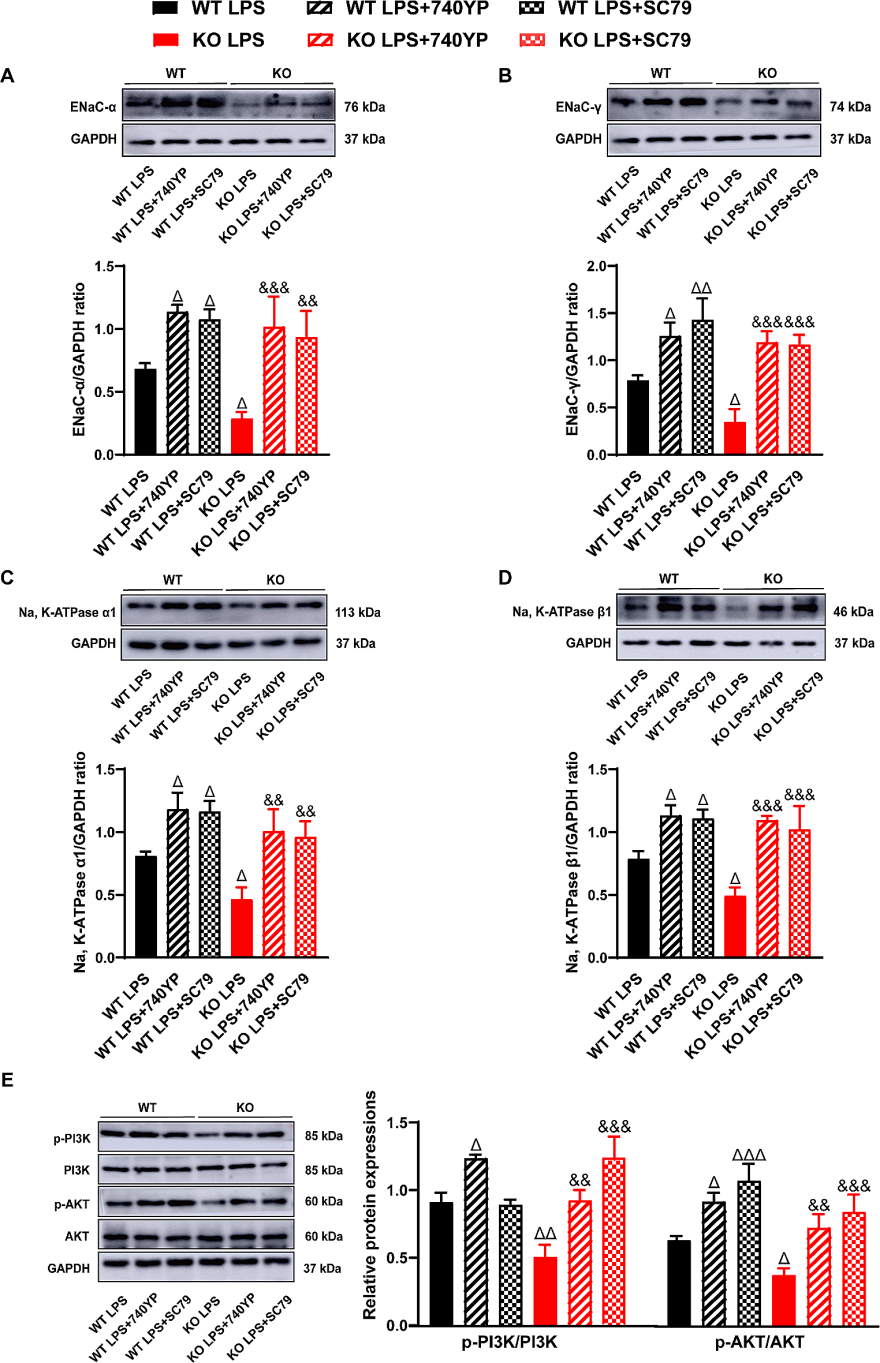
Activated PI3K/AKT signaling partially reversed the inhibition of PEBP4 deficiency on AFC and PI3K/AKT pathway in LPS-induced ALI. Western blotting was employed to examine the expressions of ENaC-α, γ, Na, K-ATPase α1, β1, p-PI3K and p-AKT (*n* = 3). Data are presented as mean ± SD values. ^Δ^*p* < 0.05, ^ΔΔ^*p* < 0.01 and ^ΔΔΔ^*p* < 0.001 vs. the WT LPS group; ^&&^*p* < 0.01 and ^&&&^*p* < 0.001 compared to the KO LPS group

Furthermore, we studied the effects of 740YP and SC79 on pulmonary fluid clearance and the PI3K/AKT signaling pathway in LPS-induced ALI. As expected, western blotting showed that compared with the LPS alone groups, the expression levels of ENaC-α, ENaC-γ, Na, K-ATPase α1, and Na, K-ATPase β1 proteins were significantly increased in the groups of 740YP and SC79 combined with LPS (Fig. 5A-D). Similarly, the protein expression levels of p-PI3K and p-AKT in the groups of 740YP and SC79 together with LPS were higher than those in LPS alone groups (Fig. 5E).

## Discussion

Acute lung injury (ALI) is pathologically characterized by diffuse alveolar damage, pulmonary edema, and extensive inflammatory cell infiltration [22, 23]. If severe, ALI can progress to acute respiratory distress syndrome (ARDS), a fatal respiratory syndrome. The pathogenesis of ALI is rather complicated and has not been yet fully understood. LPS, an essential cellular wall component of Gram-negative bacterium, is the most potent cause of lung injury in clinical practice, and its intratracheal administration has been widely accepted as a clinically relevant ALI model [24]. In this study, we successfully established an LPS-induced ALI mouse model and found that the expression of PEBP4, a secreted protein with a variety of biological functions [8, 9], in lung tissue was down-regulated after LPS treatment (Fig. 1A), suggesting that PEBP4 might be involved in the development of ALI.

Previous studies found that knocking out PEBP4 could aggravate acute liver injury induced by LPS/D-GalN and liver fibrosis induced by CCl4 through the NF-κB signaling pathway [11, 12], indicating PEBP4 may be a candidate regulator for inflammatory disorders. In lung diseases, available research on PEBP4 mainly focuses on lung cancer [17, 25–27], yet the influence of PEBP4 on ALI has not been studied. Hence, we constructed a PEBP4 KO mouse model (Fig. 1B-D), and observed that PEBP4 deficient mice displayed increased susceptibility to LPS-induced ALI, demonstrated by more serious lung damage and edema, and more inflammatory cell infiltration (Fig. 2A). The aggravated effect of PEBP4 KO was also observed by lung CT imaging (Fig. 2B). Their relevant grades further confirmed the above phenomenon (Fig. 2C-D). As previously mentioned, lung edema is the other central event of ALI [6]. During the process of ALI, the damage to the alveolar epithelial-endothelial barrier can result in pulmonary edema. Thus, we evaluated the W/D ratio of lung and protein concentration in the BALF, two indicators of lung edema [15]. As expected, both lung W/D ratio and BALF protein concentration were higher in PEBP4 KO mice with LPS treatment (Fig. 2E-F). Inflammatory response is another major influencing factor in the development of ALI. Diverse pro-inflammatory cytokines, such as TNF-α, IL-1β, IL-8, and IL-6, are released, and then neutrophil infiltration and activation are triggered by these cytokines, which are seen as important events in the inflammation of LPS-induced ALI [28]. Similar to this viewpoint, PEBP4 deficiency significantly increased lung MPO activity (Fig. 2G), an indicator of neutrophil infiltration [12], and promoted the secretion of serum TNF-α, IL-1β, and IL-6 (Fig. 2H-J), as well as notablely up-regulated the protein expression of lung COX-2 (Fig. 2K), an inflammatory enzyme [12], in ALI mice. Taken together, our data strongly suggest that PEBP4 represents a novel modulator of ALI progression.

What is the molecular mechanism underlying PEBP4-regulating ALI? As is well-known, the transport of Na^+^ is crucial for the clearance of alveolar fluid. Na^+^ transporters including ENaC and Na, K-ATPase are responsible for sodium ion transport [6]. The dysfunction of ENaC or Na, K-ATPase impairs AFC which can cause the occurrence of lung edema [29–31]. Mice with ENaC-α KO were proved to exhibit severe ARDS at birth and even to die within 40 h after birth due to the inability to remove fluid from the lungs [31]. Up-regulation of ENaC expression was demonstrated to promote AFC and to alleviate lung edema in animal models of ALI [32, 33]. It is worth noting that there is a closely relationship between serum PEBP4 levels and sodium ion concentration [14]. In this study, after PEBP4 KO, the expression of ENaC-α and ENaC-γ significantly decreased in ALI (Fig. 3A, C), while the ENaC-βsubunit unexpectedly showed no significant difference (Fig. 3B). This result further corroborates previous studies [34]. Similarly, the protein expression levels of Na, K-ATPase α1 and Na, K-ATPase β1 were significantly inhibited by PEBP4 KO in ALI (Fig. 3D-E). These results suggest that PEBP4 might regulate the Na^+^ transporters to influence AFC in ALI.

Which signaling might mediate PEBP4-modulated ALI and AFC? PI3K/AKT signaling is known as an important pathway in the occurrence and development of ALI [15, 35–37]. Coincidentally, a few studies have demonstrated PEBP4 has a tightly connection with PI3K/AKT pathway. On the one hand, PEBP4 was confirmed to be as a scaffold protein for AKT and mTOR to enhance their interaction and to promote the activation of AKT and mTOR [16]. On the other hand, PEBP4 was found to positively regulate the phosphorylation of AKT in various cancer models [8, 17]. Indeed, our data also revealed that the absence of PEBP4 further inhibited the activation of the PI3K/AKT signaling pathway in ALI model (Fig. 3F). Further, we verified the aggravation of PEBP4 loss to ALI, involving the changes of morphology, the degree of lung edema, and the intensity of inflammatory responses, was partly rectified by PI3K/AKT activator 740YP or SC79 (Fig. 4). At the same time, PI3K/AKT pathway was reported to involve in the process of AFC of ALI models [15, 35–37]. For instance, activation of PI3K signaling was reported to increase AFC associated with ENaC activity in a rat model [36]. It is speculated that PEBP4 might regulate AFC via PI3K/AKT pathway. As shown in this study, the expression of ENaC-α and ENaC-γ, and Na, K-ATPase in ALI with PEBP4 KO partially reversed by 740YP or SC79 as well, concomitant with further increase of both p-PI3K and p-AKT levels (Fig. 5). Collectively, this study highly supports that PI3K/AKT signaling pathway specifically mediates the promotion of PEBP4 absence in AFC and ALI.

## Conclusion

**Fig. 6:**
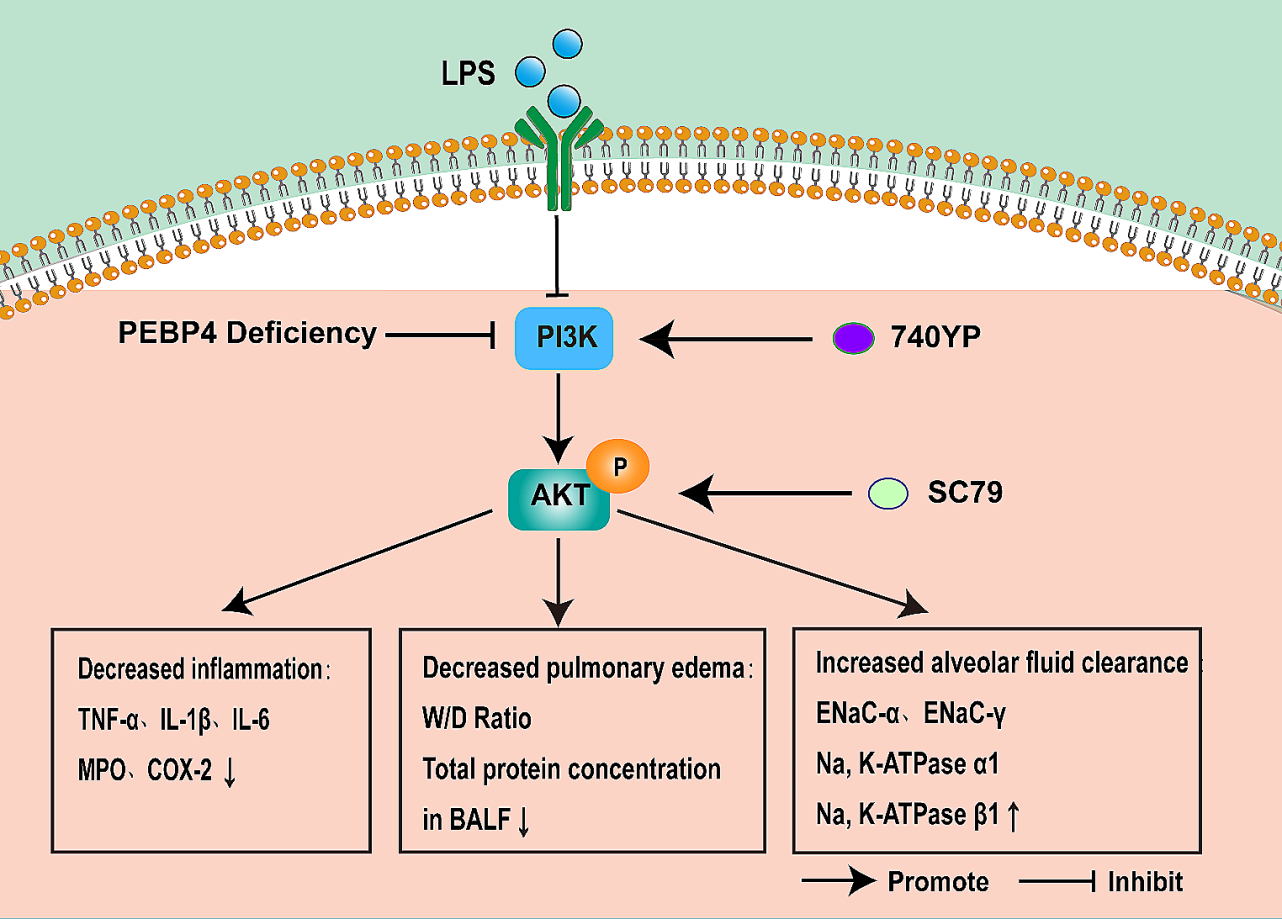
Schematic of PEBP4-regulating PI3K/AKT signaling pathway in LPS-induced ALI. PEBP4 KO exacerbated the inhibition of the PI3K/AKT signaling pathway, and then reduce AFC, and promote inflammation and pulmonary edema to aggravate ALI induced by LPS. PI3K activator (740YP) and AKT activator (SC79) partially rectified these effects

Our research applied LPS-induced ALI and PEBP4 KO models to highly demonstrated that PEBP4 deficiency can aggravate ALI and AFC, and this function may be partly mediated specifically by PI3K/AKT signaling pathway (Fig. 6), which will provide a foundation for PEBP4 as the target for ALI. Nevertheless, this study has some limitations that need to be further explored. For instance, what are the details on the molecular mechanisms by which PEBP4 regulates PI3K/AKT? Why does the administration of the PI3K/AKT activators only partially rescue the effect of PEBP4? Reports showed that many signaling pathways were involved in the regulation of ALI, such as Nrf2/HO-1 [38], TLR4/NF-κB [39], and PPARγ [40], and so on. Meanwhile, PEBP4 was also demonstrated to modulate diverse signaling pathway, such as NF-κB [11, 12], mTOR [16], AKT [17], miRNA [27]. Therefore, more studies will be conducted to determine to whether PEBP4 influences ALI via other signaling pathways and whether there is a crosstalk between these other signaling pathways and PI3K/AKT pathway. Additionally, this study lacks in evidence from specific-lung PEBP4 knockout mice, the cellular level, and clinical data, which need to be further carried out in the future work. Meanwhile, it should be noted that serum cytokine levels in this study are not the best indicator for lung inflammation. Cytokines levels in BALF should be detected in further research. Thus, it must be admitted that a PEBP4-targeted strategy applied in ALI treatment still encounters great challenges. The present findings just provided a ray of dawn that PEBP4 is an important participant in ALI progression.

## Data Availability

All original data are available as requested.
